# *p*-Hydroxyphenyl-pyranoanthocyanins: An Experimental and Theoretical Investigation of Their Acid—Base Properties and Molecular Interactions

**DOI:** 10.3390/ijms17111842

**Published:** 2016-11-05

**Authors:** Anna Vallverdú-Queralt, Michal Biler, Emmanuelle Meudec, Christine Le Guernevé, Aude Vernhet, Jean-Paul Mazauric, Jean-Luc Legras, Michèle Loonis, Patrick Trouillas, Véronique Cheynier, Olivier Dangles

**Affiliations:** 1 Institut National de la Recherche Agronomique (INRA), UMR1083 Sciences pour l’œnologie, 2 place Pierre Viala, 34060 Montpellier CEDEX, France; avallverdu@ub.edu (A.V.-Q.); meudec@supagro.inra.fr (E.M.); leguerneve@supagro.inra.fr (C.L.G.); vernhet@supagro.inra.fr (A.V.); mazauric@supagro.inra.fr (J.-P.M.); jean-luc.legras@inra.fr (J.-L.L.); 2 CIBER Fisiopatología de la Obesidad y la Nutrición (CIBEROBN), Instituto de Salud Carlos III, 28903 Madrid, Spain; 3 INSERM UMR 850, University of Limoges, School of Pharmacy, 2 rue du Dr. Marcland, F-87025 Limoges, France; michal.biler@seznam.cz (M.B.); patrick.trouillas@unilim.fr (P.T.); 4 Department of Biophysics, Centre of the Region Haná for Biotechnological and Agricultural Research, Palacký University, Šlechtitelů 27, 78371 Olomouc, Czech Republic; 5 INRA, UMR408 SQPOV, University of Avignon, 84000 Avignon, France; michele.loonis@inra.fr (M.L.); olivier.dangles@univ-avignon.fr (O.D.); 6 Regional Centre of Advanced Technologies and Materials, Department of Physical Chemistry, Faculty of Science, Palacký University Olomouc, tř. 17. listopadu 12, 77146 Olomouc, Czech Republic

**Keywords:** pyranoanthocyanins, acid-base, self-association, copigmentation, metal complexation, UV-visible absorption

## Abstract

The physicochemical properties of the wine pigments catechyl-pyranomalvidin-3-*O*-glucoside (PA1) and guaiacyl-pyranomalvidin-3-*O*-glucoside (PA2) are extensively revisited using ultraviolet (UV)-visible spectroscopy, dynamic light scattering (DLS) and quantum chemistry density functional theory (DFT) calculations. In mildly acidic aqueous solution, each cationic pigment undergoes regioselective deprotonation to form a single neutral quinonoid base and water addition appears negligible. Above pH = 4, both PA1 and PA2 become prone to aggregation, which is manifested by the slow build-up of broad absorption bands at longer wavelengths (λ ≥ 600 nm), followed in the case of PA2 by precipitation. Some phenolic copigments are able to inhibit aggregation of pyranoanthocyanins (PAs), although at large copigment/PA molar ratios. Thus, chlorogenic acid can dissociate PA1 aggregates while catechin is inactive. With PA2, both chlorogenic acid and catechin are able to prevent precipitation but not self-association. Calculations confirmed that the noncovalent dimerization of PAs is stronger with the neutral base than with the cation and also stronger than π–π stacking of PAs to chlorogenic acid (copigmentation). For each type of complex, the most stable conformation could be obtained. Finally, PA1 can also bind hard metal ions such as Al^3+^ and Fe^3+^ and the corresponding chelates are less prone to self-association.

## 1. Introduction

Among flavonoids, anthocyanins are responsible for plant pigmentation and express a variety of colors from red to blue.

In highly acidic aqueous solution (pH < 2), the red flavylium ion AH^+^ is the only anthocyanin form. As pH increases, water addition at C2 (hydration) becomes thermodynamically favorable, leading to the colorless hemiketal (in equilibrium with low concentration of pale-yellow chalcones). Around pH 4, fast deprotonation of AH^+^ competes with water addition, giving rise to the blue-purple quinonoid base A. Nevertheless, at equilibrium, A is in much lower concentration than the hemiketal, so that the solution is almost colorless. Around pH 4, deprotonation of AH^+^ also takes place, giving rise to blue-purple quinonoid bases (A, kinetic products), which are in low concentration at equilibrium. However, in their natural medium, the anthocyanin colored forms are prone to form noncovalent assemblies, mainly by π–π stacking interactions, either: (i) with themselves (self-association); or (ii) with the pool of colorless polyphenols especially, flavones, flavonols, and hydroxycinnamic acids (copigmentation) [1,2,3]. Copigmentation prevents or limits water addition, thereby maintaining higher concentrations of colored forms under mildly acidic to neutral conditions [4,5]. It is typically evidenced by a hyperchromic shift in the visible absorption band.

**Figure 1:**
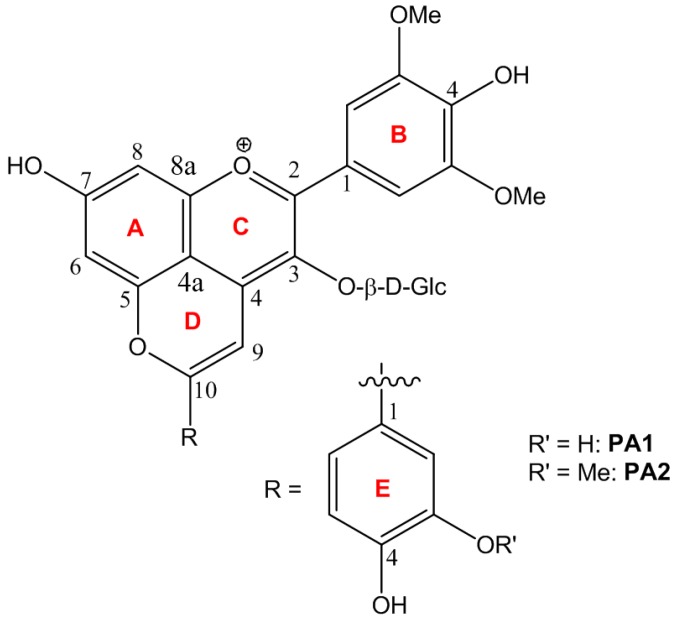
Structures of pyranomalvidins pigments catechyl-pyranomalvidin-3-*O*-glucoside (PA1) and guaiacyl-pyranomalvidin-3-*O*-glucoside (PA2).

During wine aging, anthocyanins are progressively converted to specific pigments, in particular pyranoanthocyanins with particular chromatic and physicochemical properties. Among them, *p*-hydroxyphenyl-pyranoanthocyanins result from reaction of anthocyanins with either vinylphenols [6] or hydroxycinnamic acids [7], the latter being the precursor of the former by decarboxylation. For instance, catechol and guaiacol are formed by decarboxylation of caffeic and ferulic acids, respectively, and react with malvidin-3-*O*-glucoside to form the catechyl-(PA1) and guaiacyl-(PA2) pyranomalvidin-3-*O*-glucoside derivatives (Figure 1). A detailed description of pyranoanthocyanin formation from malvidin 3-*O*-glucoside and vinylguaiacol has recently been provided, including the full characterization of the reaction medium by a combination of Liquid chromatography coupled to diode array detection and mass spectrometry (LC-DAD-MS) and accurate quantification of the major compounds by quantitative nuclear magnetic ressonance (qNMR) [8].

Controversial results have been reported on the physicochemical properties of pyranoanthocyanins. Quijada-Morín et al. [9] investigated the physicochemical and chromatic features of PA1 and PA2 in the pH range 2.0–4.5. A correct curve-fitting of the visible absorbance vs. pH curves was obtained assuming noncovalent dimerization of PA1 and two successive hydration reaction steps with no proton loss. In contrast, PA2 appeared virtually insensitive to water addition. Consistently, Oliveira et al. [10] proposed that homologues of vitisin A (R = CO_2_H, Figure 1) do not undergo hydration in the pH range 0–8 but only deprotonation reactions. Cruz et al. [11] studied four flavanyl-pyranoanthocyanins and two catechyl-pyranoanthocyanins, including PA1, in hydroalcoholic solutions. With these compounds again, only deprotonation, not hydration, was observed. However, a slow and irreversible reaction of vitisin A with water in oxidative conditions leading to neutral pyranone-anthocyanins (oxovitisins) was demonstrated [12]. Remarkably, a complete characterization by NMR of epimeric water adducts of 10-acetyl-pyranoanthocyanins (R = COCH, Figure 1) was reported by Gomez-Alonso et al. [13]. Bakker and Timberlake [14] suggested that vitisins A and B (R = H, Figure 1) undergo water addition. Asenstorfer et al. [15] confirmed hydration of vitisin A but proposed that the competing deprotonation leading to the neutral quinonoid base prevails in mildly acidic solution.

There is also discrepancy about the values reported for the molar absorption coefficients of PAs. Namely, the ε_max_ values of PA1 and PA2 at pH 0.8 were reported at 4500 and 3060 M^−1^·cm^−1^, respectively [9], whereas an earlier estimation for PA1 at pH 1.5 gave 12,200 M^−1^·cm^−1^ [16].

The aim of this work is to revisit the chromatic and physicochemical properties of *p*-hydroxyphenyl-pyranoanthocyanins (PA1 and PA2) with a specific emphasis on: (a) proton transfer vs. hydration reactions; and (b) self-association vs. copigmentation. The experimental data by UV-visible spectroscopy and dynamic light scattering (DLS) are extensively analyzed and discussed with help of quantum chemical density functional theory (DFT) calculations.

## 2. Results and Discussion

### 2.1. Characterization of PA1 and PA2

After the purification steps, PA1 and PA2 were individually analyzed by LC-DAD/ESI-MS in positive-ion mode. PA1 (λ_max_ = 506 nm) and PA2 (λ_max_ = 506 nm) were detected at *m/z* 625 and *m/z* 639, respectively. Full ^1^H and ^13^C NMR characterization confirmed the structures of both pigments (Table S1 and Table S2).

### 2.2. Structural Transformations

#### 2.2.1. UV-Visible Spectroscopy

The ε_max_ values in acidified MeOH (0.1 HCl) were measured at 18 × 10^3^ and 42.5 × 10^3^ M^−1^·cm^−1^ (λ_max_ = 520 nm) for PA1 and PA2, respectively. Those ε_max_ values are much higher than those previously reported in the literature, thereby attesting the high purity of the PA samples used in this work.

**Figure 2:**
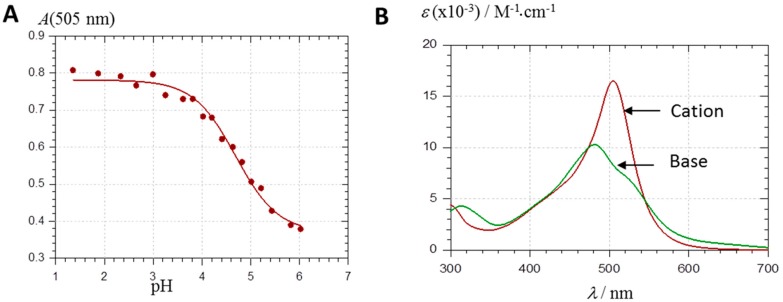
(**A**) Plot of the visible absorbance of PA1 (48.4 µM, citrate-phosphate buffer) at 505 nm (collected immediately after addition of PA1 to buffer) as a function of pH. The solid line is the result of the curve-fitting assuming a single proton transfer: p*K*_a_ = 4.69 (±0.06), rA = εA/εAH^+^ = 0.47 (±0.02), r = 0.993; (**B**) normalized spectra of pure cation (pH 1.88) and pure base (spectrum at pH 4.64 after subtraction of ca. 50% of the residual cation).

Small volumes of concentrated PA solutions in acidified MeOH (100% flavylium) were added to buffered aqueous solutions in the pH range 1–6 and the spectral properties were monitored over 15–30 min. Under such conditions, common anthocyanins undergo a typical color loss, reflecting water addition onto AH^+^ [17,18] during the period of time required to reach pseudo-equilibrium (without taking into account the slow *cis–trans* chalcone isomerization), i.e., from a few seconds (pH 2–3) to several minutes (pH 5–6). The apparent kinetics of hydration is largely governed by the fraction of AH^+^, the sole colored form to undergo water addition in solution. Unlike common anthocyanins, PA1 and PA2 do not clearly undergo such relatively fast color loss in agreement with previous investigations on various PAs [11,19]. Thus far, the sole PAs whose cationic form has been clearly shown to be electrophilic enough to add water (at C10) are the acetylpyranoanthocyanins (stemming from the addition of diacetyl-2,3-butanedione to anthocyanins) owing to the electron-withdrawing effect of the acetyl group [20]. By contrast, the visible spectra of PA1 and PA2 in the pH range 2.0–3.5 are essentially stable over time and identical to the spectra under highly acidic conditions (pH = 1), in which AH^+^ is the sole species. Above pH = 3.5, an instantaneous broadening and weakening of the visible band becomes observable with both PAs, which reflects proton loss. Therefore, a few seconds after mixing, the pH dependence of the visible absorbance at 505 nm (maximal absorption wavelength of AH^+^) can be safely analyzed with the assumption of a single proton loss. In an acetate buffer containing 12% EtOH, the first p*K*_a_ of PA1 (concentration. 50 µM) and PA2 (ca. 25 µM) was estimated at 4.30 (±0.09) and 4.35 (±0.11), respectively. In the citrate-phosphate buffer (no EtOH), the first p*K*_a_ of PA1 was estimated at 4.69 (±0.06) (Figure 2A). These values are consistent with those from the literature on PAs [11,18] and common anthocyanins [18].

The spectrum of the pure neutral base A (Figure 2B) was deduced from the spectrum obtained at pH ≈ p*K*_a_ (50% flavylium + 50% base) and the spectrum of the pure AH^+^ form (pH ≈ 2). This deduced spectrum was used as the experimental control for calculations aimed at determining the distribution of the different tautomers (deprotonation of the acidic OH groups at C4B-OH, C4E-OH or C7A-OH) and their individual visible spectra (see below). Surprisingly, the experimental spectrum of A displays a main absorption band at λ_max_ ≈ 480 nm, which means a hypsochromic shift of more than 20 nm with respect to AH^+^, and a shoulder in the range 530–540 nm. Its maximum molar absorption coefficient is circa half that of AH^+^. This uncommon hypsochromic shift is consistent with previous observations [11,19]. This could explain why hydration (formation of the colorless forms) and proton loss are not easily distinguished with PAs in absence of a careful kinetic investigation.

#### 2.2.2. DFT-Based Rationalization

**Figure 3:**
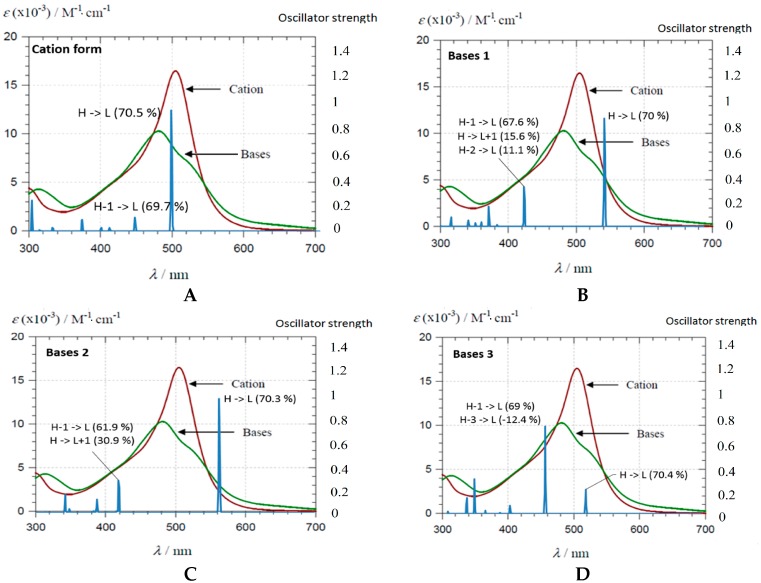
Experimental and theoretical (TD-DFT) UV-visible absorption spectra of PA1 for: (**A**) AH^+^; and the three A forms resulting from deprotonation of: C4E-OH (**B**, base 1); C4B-OH (**C**, base 2); and C7A-OH (**D**, base 3).

The AH^+^ form of PA1 exhibits a large band at 506 nm (Figure 2B), which is predicted by the TD-DFT calculation as corresponding to the first excited state S0 → S1 (at 496.3 nm, see Figure 3A).

**Figure 4:**
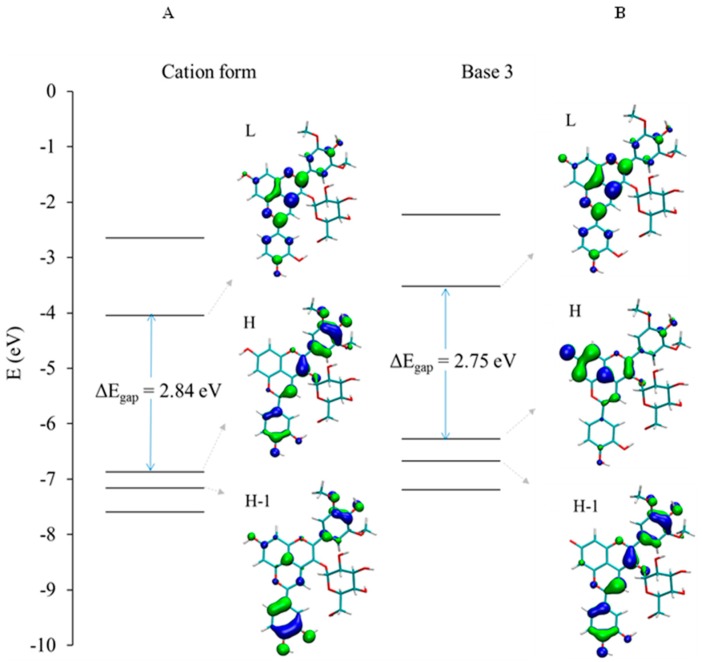
Molecular orbitals (MO) correlation diagram of PA1 for: (**A**) AH^+^; and (**B**) the A-form resulting from deprotonation of C7A-OH (base 3).

This excited state is mainly described by the HOMO → LUMO electronic transition, both molecular orbitals (MO) being delocalized along an extended conjugated path (Figure 4A).

The shoulder experimentally observed at 430 nm is ascribed to the second (S0 → S2) excited state theoretically lying at 446.4 nm (Figure 3A), which is mainly described by the HOMO-1 → LUMO electronic transition and mainly involving the E-ring, i.e., catechol moiety in the case of PA1 (Figure 4A). When increasing pH, the UV-visible absorption properties are modified, highlighting a clear hypsochromic shift (Figure 2B). The excited states of the deprotonated forms were evaluated for the three possible neutral quinonoid bases A. Concerning the first two deprotonated forms, i.e., proton loss from C4E-OH (base 1) or C4B-OH (base 2), a clear bathochromic shift is predicted for the first excited state (Figure 3B,C). In both cases, this excited state is still described by the HOMO → LUMO electronic transition. Whereas the LUMO is almost identical for AH^+^ and bases 1 and 2, deprotonation of C4E-OH or C4B-OH induces a destabilization of the HOMO (Figure S1A,B), therefore decreasing the energy gap and resulting in a bathochromic shift. By contrast, upon deprotonation of C7A-OH (base 3), the HOMO is dramatically displaced toward the A-ring, therefore strongly decreasing the overlap with the LUMO. As a consequence, the first excited state (S0 → S1) at 517.9 nm has relatively low oscillator strength compared to AH^+^ (0.2 and 0.9, respectively) and is slightly bathochromically shifted (as for bases 1 and 2) (Figure 3D). However, HOMO-1 is moved toward the C- and D-rings (Figure 4B), thus increasing the overlap with the LUMO and the oscillator strength of the S0 → S2 excited state at 454.1 nm. In conclusion, the observed hypsochromic shift is not an actual shift but an inversion in intensity between the first and second excited states. This is confirmed by the presence of a shoulder experimentally observed at 540 nm (Figure 3D). The agreement between the theoretical and experimental spectra supports the presence in solution of only one neutral quinonoid base corresponding to proton loss from C7A-OH. This is confirmed by the relative free enthalpies of the three quinonoid bases, showing that base 3 is energetically preferred by ca. 3 kcal·mol^−1^ (Table 1).

According to the Boltzmann law, each minor quinonoid base (bases 1 and 2) represents only 1% of the total; their presence most probably contributes only slightly to the shoulder observed at 540 nm. The same rationalization also applies to PA2 (see Table S3 and Table S4).

### 2.3. Noncovalent Assemblies of Pyranoanthocyanins

#### 2.3.1. Self-Association

Self-association of anthocyanins has been investigated by UV-visible spectroscopy, circular dichroism and NMR [21,22,23]. At high concentration (>0.1 mM), anthocyanins typically form left-handed aggregates, which results in relatively modest modifications in the visible spectra (band broadening with a slight decrease of absorption at λ_max_). Interestingly, investigations by ^1^H-NMR in acidic to mildly alkaline solutions [21,22] gave the following values for the dimerization constants of the colored forms of malvidin 3,5-diglucoside: AH^+^: 600, A: 900, A^−^: 200 M^−1^. This means that, as expected, dimerization is stronger for the neutral form (pH 4–7), i.e., when electrostatic repulsion is minimized between the two chromophores.

By contrast, little data is available about the self-association of pyranoanthocyanins. One study was reported with the pyranoanthocyanin formed by addition of 8-vinylcatechin (itself a product of the aromatic electrophilic substitution of acetaldehyde with catechin) and malvidin 3-*O*-*β*-d-(6-*O*-*p*-coumaroyl)glucoside. At pH = 1.3, the AH^+^ form of this PA was shown to form a noncovalent dimer with a binding constant Kd of 3 × 10^3^ M^−1^ [11], which is only circa twice higher than for the parent anthocyanin (estimated in the presence of 30% DMSO and thus probably lower than its value in aqueous solution) [23]. Although the AH^+^ forms of PAs do not seem much more prone to self-association than the parent anthocyanins, under mildly acidic conditions, AH^+^···A or A···A self-association is likely to be stronger due to the decrease in electrostatic repulsion.

**Figure 5:**
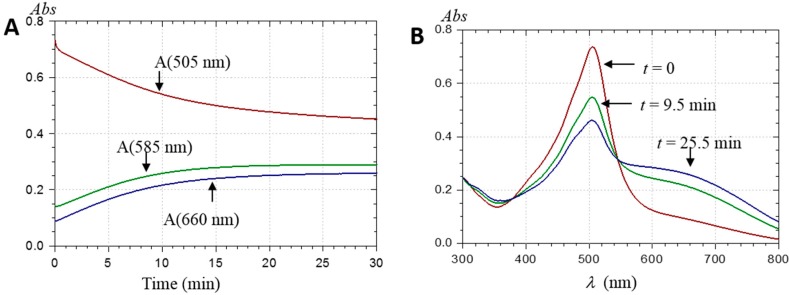
(**A**) Changes in visible absorbance (*Abs*) following the addition of PA1 (48.4 µM) to a pH 4.39 acetate buffer + 12% EtOH; and (**B**) UV-visible spectra at different time points.

**Figure 6:**
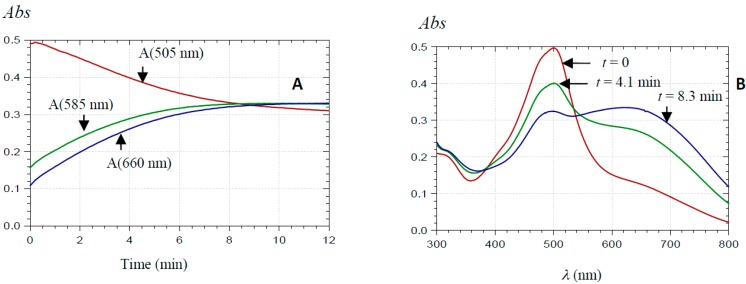
(**A**) Changes in visible absorbance following the addition of PA1 (48.4 µM) to a pH 5.20 acetate buffer + 12% EtOH; and (**B**) UV-visible spectra at different time points.

In this work, a surprising observation is the slow decay of the main visible band taking place in 50 µM solutions of PA1 at pH > 3.5 (Figure 5 and Figure 6).

**Figure 7:**
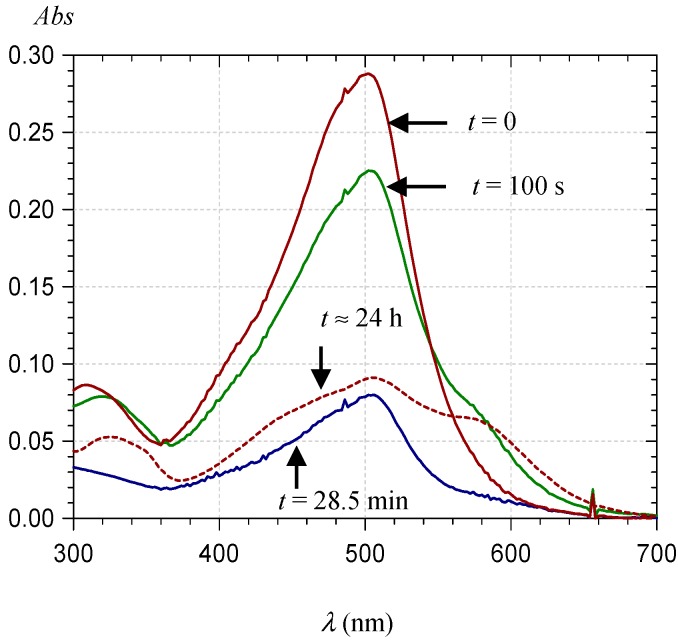
Visible spectra of PA2 (10 µM) in a pH 5.0 acetate buffer + 12% EtOH at different time (*t*) points.

These spectral changes are not consistent with hydration, being too slow, non-first-order, faster as the pH increases, and accompanied by the build-up of broad absorption bands at longer wavelengths (λ ≥ 600 nm). To the best of our knowledge, such observations have never been reported before. A reasonable explanation for the slow changes observed in the visible spectra of PA1 at pH > 3.5 is that self-association involving the neutral quinonoid base occurs. Mixed aggregates AH^+^···A may form first, followed by aggregates of pure base A at higher pH, which is consistent with the spectral differences between pH 4.4 (Figure 5) and pH 5.2 (Figure 6). The additional methyl group should make PA2 (25 µM) more prone to self-association and precipitation than PA1 (50 µM). As a direct consequence of more favored noncovalent interactions, precipitation rapidly occurred in PA2 solutions at pH > 4 (even in presence of 12% EtOH), despite relatively low concentration. By contrast, PA1 solutions remained free of precipitate over hours in the presence of 12% EtOH. Similar observations were made in dilute (10 µM) PA2 solutions. Unlike PA1, aggregation in dilute PA2 solution, which occurred relatively rapidly at pH > 4.5, was mainly manifested by a drop of visible absorbance and low-energy absorption bands were much weaker than with PA1 (Figure 7).

The higher propensity of A for self-association is corroborated by the concentration dependence of the first p*K*_a_ value. Indeed, dilution by a factor of 2.5 shifted p*K*_a_ value from 4.35 (±0.11) to 4.79 (±0.12) for PA2. This means that at high concentration, proton loss occurs at lower pH (acid-base equilibrium shifted toward the base) in agreement with A being more prone to self-association than AH^+^.

DFT calculations including dispersion correction confirmed the capacity of PA1 and PA2 to self-associate, both exhibiting negative energies of association (Table 2 and Table S3 for PA1 and PA2, respectively).

**Figure 8:**
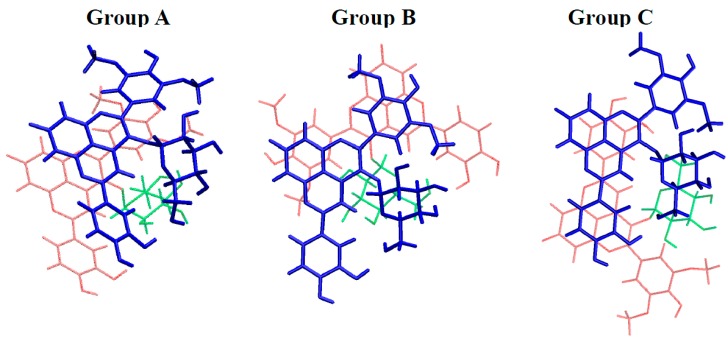
Three representative conformers of PA1 self-association complexes of the three representative groups, exhibiting geometrical similarities: Group (**A**) interactions between A-, C-, and D-rings of both PA1 fragments; Group (**B**) interactions between A-, C-, and D-rings of one PA1 and the B-ring of the other PA1; and Group (**C**) interactions between A-, C-, and D-rings of one PA1 with the E-ring of the other PA1. For clarity purpose, one PA1 partner is in blue, whereas the other is both in red (pyranoanthocyanidin moiety) and in green (sugar moiety).

The three possible self-associations (AH^+^···AH^+^, AH^+^···A, and A···A) appeared likely to occur. For each, several conformers were obtained (see Figure 8 and Table 2 for geometrical description and stabilizing energies, respectively), in which π–π stacking is the driving force of association.

Three types of conformers could be distinguished: Group A, exhibiting interactions between A-, C-, and D-rings of both PA1 units; Group B, exhibiting interactions between A-, C-, and D-rings of one PA1 and the B-ring of the other PA1; Group C, exhibiting interactions between A-, C-, and D-rings of one PA1 with the E-ring of the other PA1. In each group, electrostatic and H-bonding interactions influence orientation of both units with respect to each other. In particular, the strong electrostatic repulsion in AH^+^···AH^+^ complexes strongly distorted the geometries (compared to AH^+^···A and A···A), however without preventing the stabilizing π–π stacking forces.

Self-association is here studied in terms of electronic energy, not Gibbs energy, mainly because entropy related to self-association cannot be accurately calculated [3]. Hence, if the most stable conformer can be regarded as the major conformer present in solution, all other conformers must also be considered, although with relative weight obtained from their relative stabilizing energy. Having this in mind, Table 2 shows that A···A association is favored as the most stable A···A conformer is more stable by ca. 3 kcal·mol^−1^ than the most stable AH^+^···A and AH^+^···AH^+^ conformers. Moreover, stable A···A conformers appear more numerous, in particular when comparing to AH^+^···AH^+^ dimers. In this latter case indeed, only one AH^+^···AH^+^ dimer exhibits a stabilizing energy close to those of the AH^+^···A dimers. Hence, DFT calculations including dispersion forces confirm that AH^+^···AH^+^ (pH < p*K*_a_) forms to a lesser extent than AH^+^···A (pH ≈ p*K*_a_) and A···A (pH > p*K*_a_), in agreement with the experimental observations.

The observation that PA2 is more prone to self-association and precipitation than PA1 was also confirmed by the dispersion corrected (DFT-D) calculations, as the most stable A···A conformers exhibited association energies of −32.3 and −28.0 kcal·mol^−1^, for PA2 and PA1, respectively (Table 2 and Table S3). As expected, replacing an OH group by an OMe group on an aromatic ring increases π–π stacking interactions by slightly extending the π-conjugated system [3].

**Figure 9:**
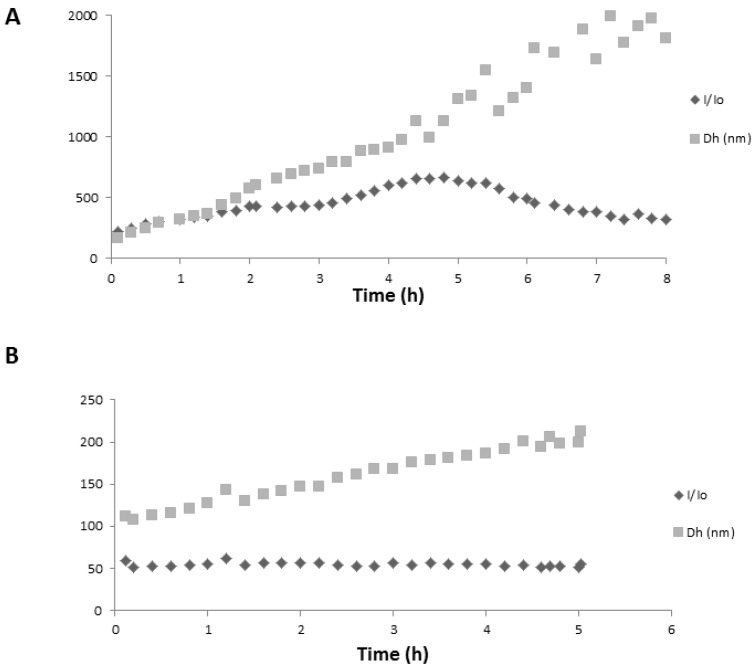
Aggregation kinetics followed by dynamic light scattering (DLS) (observation angle = 90°). A rise in the normalized scattered intensity I/I_0_ above 1 indicates the onset of aggregation. I_0_, intensity scattered by the solvent; I, intensity scattered after PA1 addition. Dh: average hydrodynamic diameter of the aggregates, weighted according to the intensity. *Y* axis indicates I/I_0_ or Dh value. (**A**) PA1 (50 µM) at pH 4.4 (+12% EtOH); and (**B**) PA1 (50 µM) with chlorogenic acid (100 equiv.) at pH 4.4 (+12% EtOH).

Noncovalent dimerization must be instantaneous (diffusion-controlled), which is in contrast to the slow spectral changes observed with PAs. It is thus very likely that PA self-association is not limited to the formation of noncovalent dimers or small-sized oligomers but leads to large aggregates. This was confirmed by DLS experiments. For PA1 (50 µM), an immediate increase in the relative scattering intensity (I/I_0_) was observed at pH 4.4 (Figure 9A) and 5.2, which was related to the immediate formation of polydispersed aggregates (PI = 0.4) with an average hydrodynamic diameter (Dh) of ca. 150 nm. With such polydisperse dispersions, it must be kept in mind that the Dh value is only indicative and in favor of the largest particles. The average Dh and polydispersity index (PI) kept increasing during the experiment, up to the formation of micron-sized aggregates and to a PI of 1, whereas I/I_0_ reached a plateau-value after about 3–4 h. This indicated that no new aggregates formed at this stage. After 5 h, the decrease in I/I_0_ was related to precipitation of micron-sized aggregates (Figure 9A). At pH = 3.5, there was no change in scattering intensity, meaning that no aggregation occurred. For PA2 (25 µM), precipitation rapidly occurred after mixing while interaction kinetic was significantly slowed down at lower concentration (10 µM).

#### 2.3.2. Copigmentation

**Figure 10:**
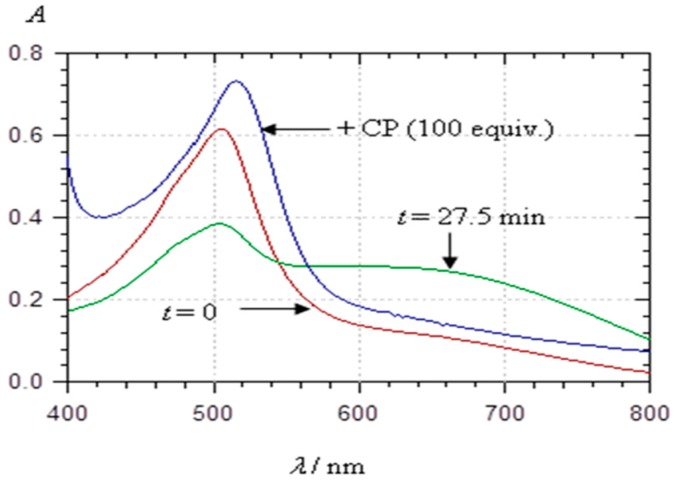
Visible spectra of PA1 (48.4 μM) in a pH 4.62 acetate buffer + 12% EtOH: immediately after addition (*t* = 0), after the development of self-association (*t* = 27.5), and after the subsequent addition of the copigment chlorogenic acid (Copigment, 100 equiv).

Adding a large excess of chlorogenic acid to PA1 aggregates (50 µM PA1 solution ca. 30 min after preparation) led to conversion of the visible spectrum back to that of a fresh solution (i.e., before the onset of aggregation) (Figure 10).

This suggests the dissociation of the PA1 aggregates due to the competitive formation of noncovalent PA1···chlorogenic acid complexes. A bathochromic shift of the λ_max_ value by ca. 10 nm was observed, which is typical of copigmentation. Copigmentation is actually known to compete with anthocyanin self-association. Moreover, the time-dependent spectral changes that were observed due to PA1 self-association at pH value of 4.6 were markedly slowed down when the chlorogenic acid concentration was increased and totally abolished with 100 equiv. copigment (data not shown). DLS experiments confirmed that addition of chlorogenic acid (100 equiv.) inhibited self-association of PA1 and prevented the formation of large aggregates prone to precipitation (in acetate buffer containing 12% ethanol at pH = 4.4 and 5.2): the scattering intensity remained unchanged and only a slight Dh increase from 100 to 200 nm was observed (Figure 9B). Unlike chlorogenic acid, catechin was unable to reverse PA1 self-association in agreement with catechin having less affinity for anthocyanins than chlorogenic acid [3].

With PA2 (25 µM), both chlorogenic acid and catechin (20–100 equiv.) were able to prevent precipitation at pH > 4 but not self-association. As said above, under more dilute conditions (10 µM), precipitation is not observed, even in the absence of copigment, and self-association is slow at pH = 4.4. However, at pH ≥ 5, self-association is much faster (Figure 7) and its kinetics is not significantly influenced by any copigment. The DLS experiments showed that addition of chlorogenic acid to PA2 (pH = 4.4 and pH ≥ 5) did not induce any change in the scattering intensity, but the aggregate size kept increasing, meaning that chlorogenic acid can indeed inhibit precipitation but not self-association (data not shown).

Theoretical calculations confirmed the formation of copigmentation complexes between chlorogenic acid and both PA1 and PA2. For PA1, complexation with AH^+^ appeared less favorable than with A (deprotonation of C7A-OH). For instance, the stabilizing energies of the most stable PA1···chlorogenic acid conformers were −18.0 and −24.5 kcal·mol^−1^ for AH^+^ and A, respectively (Table 3). This difference is weaker (1.3 kcal·mol^−1^) with PA2 (Table S3).

**Figure 11:**
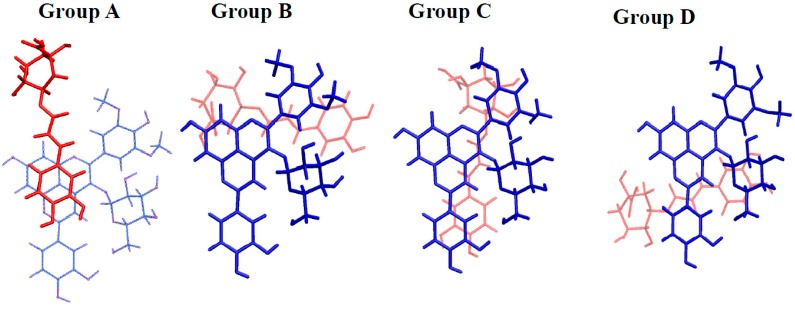
Four representative conformers of copigmentation complexes between PA1 (blue) and chlorogenic acid (red) of the four representative groups, exhibiting geometrical similarities. The catechol moiety of chlorogenic acid is in interaction with the following moieties of PA1: Group (**A**) A-, C- and D-rings; Group (**B**) B-ring; Group (**C**) E-ring; and Group (**D**) glucose.

Copigmentation association is mainly driven by π–π stacking with many possible orientations of chlorogenic acid with respect to pigment. Four groups can be distinguished in which the catechol moiety of chlorogenic acid binds the A-, C- and D-rings (Group A), the B-ring (Group B), the E-ring (Group C) or the sugar moiety (Group D) of PA (Figure 11, Figure S2 and Figure S3).

In Group D, H-bonding would mainly drive copigmentation; as expected, the corresponding conformers are less stable than those of Groups A, B, and C. In Groups A, B, and C, π–π stacking is the driving force, intermolecular electrostatic interactions and H-bonding driving only the respective orientation of pigment and copigment. Copigmentation of PAs with chlorogenic acid appears less favorable than self-association. Indeed, when comparing geometries of noncovalent dimers and copigmentation complexes, π–π stacking in noncovalent dimers is much more favored due to more extended π-conjugated systems facing each other. This is consistent with the observation that chlorogenic acid has to be used in large excess (vs. pyranoanthocyanins) to prevent aggregation. Aggregation involves the formation of dimers and higher oligomers of high conformational variability. At high concentration, copigments can intercalate in small aggregates, thus slowing down or even inhibiting the formation of larger aggregates. Copigments can also intercalate in large aggregates and promote their dissociation.

#### 2.3.3. Metal Complexation

**Figure 12:**
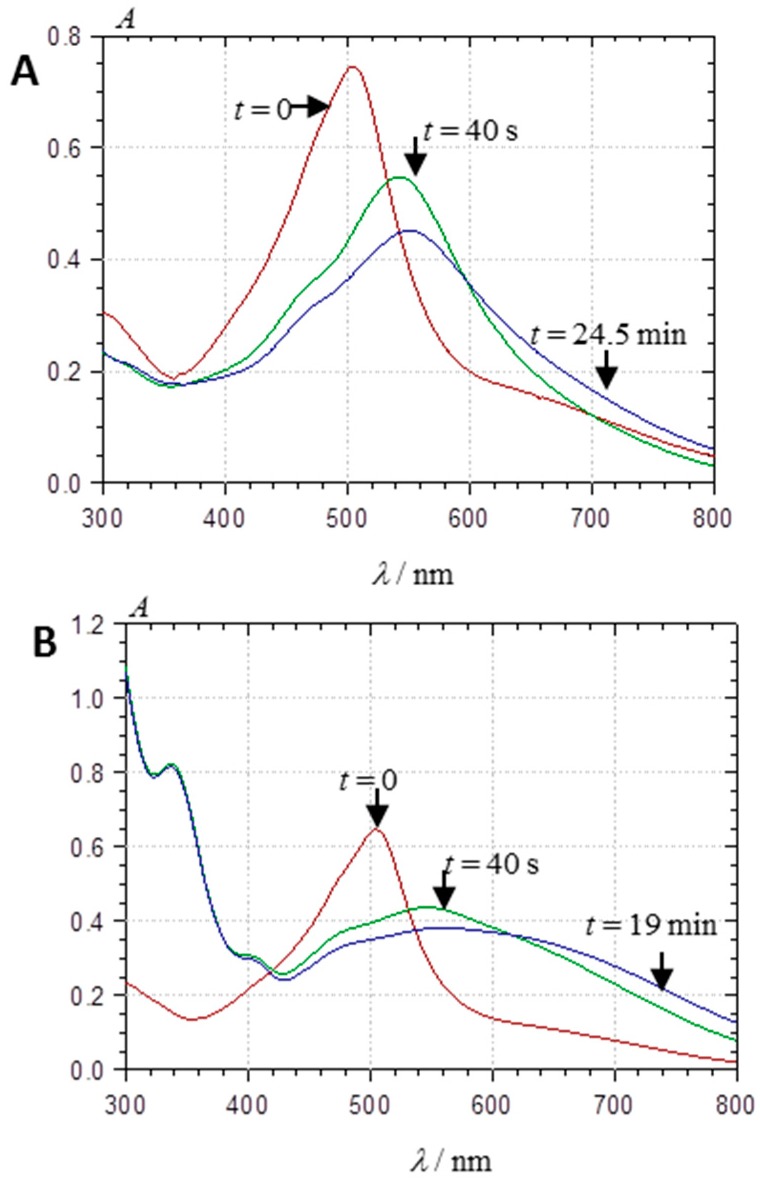
Visible spectra of PA1 (48.4 µM) in a pH 4.62 acetate buffer + 12% EtOH: immediately before metal addition and after metal addition (10 equiv.) at two time points: (**A**) Al^3+^; and (**B**) Fe^3+^.

Owing to its catechol group, PA1 is able to bind hard metal ions such as Al^3+^ and Fe^3+^. Under mildly acidic conditions, Al^3+^ binding and concomitant loss of the two catechol protons result in a strong bathochromic shift and a decrease in visible absorbance at λ_max_ (Figure 12A). With Fe^3+^, a strong band broadening is also observed, which features additional ligand-to-metal charge transfer (Figure 12B).

Both bindings are fast, being completed within 10–20 s. It is noteworthy that the metal chelates thus formed seem less prone to self-association (a possible consequence of charge repulsion) as the spectral changes observed over 20–30 min are less intense than with metal-free PA1.

### 2.4. Spectral Shifts Related to Noncovalent Association

**Figure 13:**
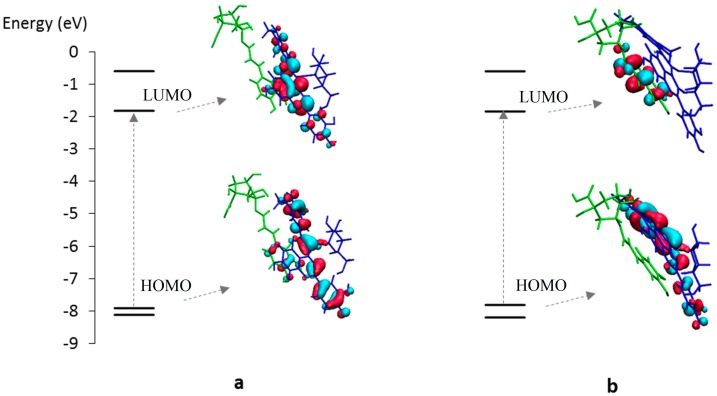
Molecular orbital description of the copigmentation complex of PA1 (flavylium ion) with chlorogenic acid: without (**a**); or with (**b**) CT character in the excited state.

The spectral shifts characteristic of self-association and copigmentation were studied by TD-DFT calculations and analyzed according to the π → π* electronic transitions constituting the first excited states (corresponding to λ_max_). Only the spectral characteristics of the AH^+^···chlorogenic acid and A···A complexes will be detailed. In the former case, λ_max_ is bathochromically shifted in agreement with the experiment. The low-energy band is assigned to the first excited state (S0 → S1), which is mainly described by HOMO → LUMO electronic transition. As already observed [3], the spectral behavior of these noncovalent complexes is highly dependent on the conformer. In some cases, the charge transfer (CT) character is strong; in some others, it is very weak (Table 4 and Figure 13).

When CT is significant, the HOMO is on the A-, B-, C-, and E-rings of the pigment, whereas the LUMO is delocalized on the copigment (Figure 13B). The average value of the spectral shift is ca. 10 nm (Table 4). Copigmentation of the quinonoid base also induces significant bathochromic shifts, which can be even higher than for the cation (Table S5).

Self-association also leads to global bathochromic shifts. However in this case, the MO analysis is more complex. Indeed, with the A···A dimer, several excited states must be considered, most of them leading to bathochromic shifts with respect to the most intense band of the monomer. For instance, for the most stable conformer, the first four excited states correspond to bathochromic shifts of 11.5, 26.4, 41.9, and 57.9 nm, for S0 → S4 to S0 → S1, respectively (Table 5).

**Figure 14:**
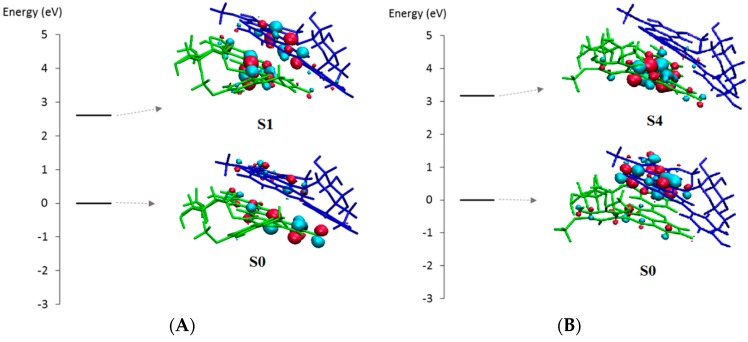
Molecular orbital description of the A···A PA1 dimer: (**A**) Geometry 1 exhibiting slight CT in the first excited state (S0 → S1); and (**B**) Geometry 4 exhibiting strong CT in the fourth excited state (S0 → S4).

This leads to four visible absorption bands of different intensity and CT character (Figure 14).

Considering the multitude of conformers, the noncovalent dimer is optically characterized by a multitude of bathochromically shifted absorption bands. In the situation of small n-mers or large aggregates, this effect is expected to increase, i.e., multiplication of conformers and of bathochromically shifted excited states. This is consistent with the very broad low-energy visible absorption bands gradually appearing in the experimental PA spectra recorded in mildly acidic conditions (Figure 5 and Figure 6).

## 3. Materials and Methods

### 3.1. Chemicals

All solvents were of HPLC quality and all chemicals of p.a. grade. Vinylguaiacol and glycerol were purchased from Sigma (Saint-Louis, MO, USA). Malvidin-3-*O*-glucoside chloride, *p*-coumaric, chlorogenic and caffeic acids were purchased from Extrasynthèse (Genay, France). Purified deionized water (MilliQ purification system, Millipore, Molsheim, France) was used for the preparation of all solutions.

### 3.2. Production of Vinylcatechol

Vinylcatechol was obtained from caffeic acid by bioconversion using the yeast strain *Saccharomyces cerevisiae* MTF3686 (internal collection). A two liters cell culture was prepared as described earlier [24] with some modifications: glycerol 10 g/L was used as a carbon source for the YNB media, and 0.5 mM of *p*-coumaric acid was added to induce the decarboxylase activity. Cells were recovered in exponential growth phase, washed twice with 50 mM citrate-phosphate buffer (pH = 3.5). Highly dense suspensions of resting cells were prepared in 1 L of citrate-phosphate buffer (pH = 3.5) containing 2% glucose and supplemented with 0.5 mM of caffeic acid as a bioconversion substrate. The medium was agitated at 27 °C during 4 days, then centrifuged (1000× g) for 10 min. The supernatant was passed through a Sep-Pak^®^ tC18 cartridge (10 g reversed-phase sorbent, Waters, Milford, MA, USA), eluted successively with H_2_O and MeOH. The MeOH fraction containing vinylcatechol was mixed with water and concentrated under vacuum to remove MeOH, analysed by HPLC-DAD as described earlier [8] to determine the concentration as equivalent vinylguaiacol, and used for the synthesis immediately.

### 3.3. Pyranoanthocyanin Synthesis

Malvidin-3-*O*-glucoside (2 mM) and vinylguaiacol (2 mM) or vinylcatechol (2 mM equiv. vinylguaiacol) were prepared in MeOH/H_2_O mixture (70/30). The pH of the mixture was adjusted with concentrated HCl to pH 3. The reactions were carried out during 5 days at 35 °C [8]. The reaction media were concentrated and re-dissolved in 1% HCOOH in H_2_O.

### 3.4. Purification of PA1 and PA2

Semi-preparative purification of PA1 and PA2 was performed using a Varian LC 940 system (Agilent Technologies, Santa Clara, CA, USA) with a UV detector and fraction collector. After filtration on a 0.45 µm HVLP Millipore filter, the solution was injected onto a Microsorb 100—3 µm, C18 column (100 × 21.4 mm, Dynamax, Santa Clara, CA, USA). Elution was achieved at a flow rate of 10 mL/min with solvents A (0.1% aqueous HCl) and B (0.1% HCl in MeCN-H2O (4:1)) with the following gradient: 0–6 min, linear 39%–41% B, 6–8 min, linear 41%–80% B, 8–9 min, isocratic 80% B, 9–10 min, linear 80%–39% B. The collected fractions were freeze-dried and stored at −80 °C until analysis.

### 3.5. NMR Analysis

All experiments were performed on an Agilent 500 MHz DD2 NMR spectrometer (Agilent Technologies, Santa Clara, CA, USA) operating at 500.05 MHz and 125.75 MHz for ^1^H and ^13^C, respectively, and equipped with a 5 mm indirect detection Z-gradient probe. The chemical shifts were expressed with MeOH as the internal reference (*δ* = 3.31 and 49.1 ppm for ^1^H and ^13^C, respectively). All data were collected with active sample temperature regulation. Assignments of proton and carbon signals were deduced from homonuclear ^1^H correlation spectroscopy, rotating-frame overhauser spectroscopy, diffusion-ordered spectroscopy and heteronuclear ^1^H/^13^C heteronuclear single quantum coherence and heteronuclear multiple bond correlation experiments. Data were processed and analyzed using the VNMRJ software (Agilent, Santa Clara, CA, USA).

### 3.6. UV-Visible Spectroscopy

Spectra were recorded with an Agilent 8453 diode array spectrophotometer fitted with a quartz cell (optical path length = 1 cm) equipped with a stirring magnet. A constant temperature of 25 °C in the cell was obtained by use of a water-thermostated bath.

Small volumes of concentrated PA solutions in acidified MeOH (0.1 M HCl, 100% flavylium) were added to a 0.2 M acetate buffer containing 12% EtOH or to a citrate-phosphate buffer (pH range 1–6). In the cell, the pigment concentration was ca. 50 µM for PA1 and 25 or 10 µM for PA2. The spectral monitoring was carried out over 15–30 min. Then, the samples were collected and kept overnight at room temperature to repeat the spectroscopic analysis. The experiments were also repeated with buffers containing a copigment (CP: chlorogenic acid or catechin) in large excess (20–100 equiv.) or a metal ion (PA1 + 10 equiv. AlCl_3_ or Fe(NO_3_)_3_) in acetate buffer + 12% EtOH.

Alternatively, some samples without CP were combined at the end of the kinetic monitoring and the pH was adjusted to 4.5 or 5.2. To the resulting solutions, the copigment was added (CP-to-PA molar ratios = 20–100) and the UV-visible spectra were recorded.

### 3.7. Dynamic Light Scattering (DLS)

DLS experiments were conducted with a Malvern Autosizer 4800 HS (Malvern Instruments, Malvern, UK) equipped with a 30 mW He-Ne laser (λ = 633 nm) and APD detection. Measurements were carried out at an angle of 90° from the incident beam. Prior to experiments, all solvents were filtered on 0.2 μm microfiltration units (Millipore, Millex-GV, Molsheim France). Both PA1 and PA2 were prepared in 0.1 M HCl in MeOH. PA solutions (10 µL) were diluted in acetate buffer (pH = 3.5, 4.4 and 5.2, 2 mL) and introduced in the measurement cell at 25 °C. Each experiment was carried out in duplicate. Control measurements were performed on solvents. Addition of chlorogenic acid (100 equiv.) to PA1 and PA2 was performed in acetate buffer containing 12% ethanol at pH = 3.5, 4.4 and 5.2.

Sample evolution was followed by measuring the scattered intensity and the autocorrelation function G(t) of the scattered light. Each measurement was the average of 10 subruns. A rise in scattered intensity above control values indicated the onset of aggregation.

The autocorrelation function of the scattered light was first analyzed using the cumulant method, which gives an average value of the aggregate size in the dispersion. The logarithm of the normalized correlation function was fitted to a polynomial:

log (G(t)/B-1) = a + bt + ct2 +…

where G(t) are the experimental correlation points, B is the baseline, and a, b, and c are the coefficients of the cumulant fit determined by a simple linear least-squares fitting procedure (a: intercept, b: slope giving access to the relaxation time of the signal). The diffusion coefficient D of the colloids was calculated with the following formula:

1/b = 2DK2

where K = (4πn_0_/λ)·sin(θ/2) is the scattering vector, n0 the refractive index of the solvent, λ the laser wavelength and θ the scattering angle. The average hydrodynamic diameter of the particles Dh was then derived from D using the Stokes-Einstein equation and assuming spherical shapes:

1/b = kT/(3π·η·Dh)

where k is the Boltzmann constant, T the temperature and η the solvent viscosity.

Finally, the polydispersity index PI (0 < PI < 1), defined as c/2b, was estimated as a measure of the variance (squared standard deviation) of the particle size distribution.

### 3.8. Methods of Calculation for UV-Visible Properties of Single Compounds

Geometry optimization were achieved using density functional theory (DFT) calculations with the hybrid functional B3P86, which has appeared particularly adapted to assess both the thermodynamic and UV-visible absorption properties of polyphenols [20,25]. The Pople-type triple-ζ basis set, namely 6-31+G(d,p), allowed to establish reliable structure-property (UV-visible absorption) relationships for large series of polyphenols at a reasonable computational time [26]. The frequency analysis, performed at the same level of theory, confirmed the absence of imaginary frequencies in the ground state (GS). Excited singlet state (ES) energies were evaluated from the GS geometries, using the time dependent (TD) DFT formalism with the same functional and basis set. The allowed vertical π → π* electronic excitation energies were thus obtained, which consequently gave the absorption energies in the UV-visible range with the contribution of all one-electron transitions as well as their oscillator strength.

The TD-DFT calculations were performed both in the gas phase and in solvent, using the Polarizable Continuum Model (PCM) scheme. The integral equation formalism PCM (IEFPCM) method coupled to UA0 radii was used. In the PCM formalism, the solute is embedded in a shape-adapted cavity surrounded by the solvent implicitly described as a dielectric continuum characterized by its dielectric constant ε (ε = 32.6 for MeOH). All calculations were performed with the Gaussian software package [27].

### 3.9. Methods of Calculation for Self-Association and Copigmentation

Self-association and copigmentation complexes were obtained from pure quantum chemistry calculations, using dispersion corrected DFT (DFT-D, D for “dispersion corrected”), which is a successful approach to evaluate noncovalent (mainly π–π stacking) interactions in copigmentation complexes. However, pure quantum chemistry methods do not allow the explicit description of solvent, especially water, for such quite large molecular systems. As a consequence, the contribution of hydrogen bonding to stabilizing energies is overestimated. Moreover, only the stabilizing electronic energies (enthalpy changes), not the free enthalpy changes, were evaluated as calculation of entropic contributions in self-association and copigmentation is still an unsolved theoretical challenge [3]. In other words, this theoretical evaluation only provides trends and not absolute ΔG of association.

All geometries of the dimerization and copigmentation assemblies were obtained with the COSMO B3P86-D2/def2-SVP formalism in the ORCA package [3]. Electronic energies were calculated with the same functional using the larger basis set def2-TZVPP. For all noncovalent complexes, binding energies were obtained as the difference in energy between the complexes and the separated partners. The geometries were used to calculate theoretical UV-visible absorption properties (spectral shifts and electronic transitions) using time-dependent DFT with IEFPCM solvent model and ωB97XD/6-31+G(d,p) formalism (Gaussian software package). The ωB97XD functional appears crucial to correctly account for possible charge transfer in the excited state.

## 4. Conclusions

In conclusion, catechyl-pyranomalvidin-3-*O*-glucoside (PA1) and guaiacyl-pyranomalvidin-3-*O*-glucoside (PA2) are stable wine pigments that do not undergo water addition and thus simply behave as monoacids in mildly acidic solution. However, in the pH range 3.5–6 that is most important for application as food colorants, their intense color tends to evolve due to self-association of the neutral quinonoid base and eventual precipitation. If controlled by appropriate matrices, possibly including some copigments to avoid precipitation, this phenomenon, reported in this work for the first time, is very interesting as natural colors from red (monomer) to blue (aggregates) can thus be produced.

## Figures and Tables

**Table 1:** Relative free energies (kcal·mol^−1^) of the quinonoid bases for pigments catechyl-pyranomalvidin-3-*O*-glucoside (PA1) and guaiacyl-pyranomalvidin-3-*O*-glucoside (PA2).

Pyranoanthocyanin Form	PA1	PA2
Base 1	4.28	3.60
Base 2	4.31	3.14
Base 3	0	0

**Table 2:** Self-association energy (kcal·mol^−1^) of PA1, according to the charge state (A···A, AH^+^···A and AH^+^···AH^+^) of the two partners and according to the geometries of the different association complexes gathered in terms of structural similarities as three different groups (A, B and C). The association energies of the most stable conformers (for the three charged forms) are highlighted in bold.

Group	Geometry	A···A	AH^+^···A	AH^+^···AH^+^
A	1	**−28.0**	−22.3	−10.1
	2	−23.2	**−25.3**	**−25.4**
	3	−22.8	−22.2	−11.4
	4	−19.2	−19.9	−9.9
	5	−18.2	−22.4	−14.9
	6	−14.9	−15.9	−9.0
	7	−12.7	−13.4	−7.9
B	8	−15.9	−21.6	−8.6
	9	−15.6	−15.3	−9.5
C	10	−17.9	−8.5	−6.0
	11	−15.2	−14.0	−10.0

**Table 3:** Association energies (kcal·mol^−1^) of PA1 (in both AH^+^ and A forms) with chlorogenic acid. Groups A, B, C and D are defined according to geometry similarity (see text and Figure 11). The association energies of the most stable conformers are highlighted in bold.

AH^+^···Chlorogenic Acid			A···Chlorogenic Acid		
Group	Geometry	Binding Energy	Group	Geometry	Binding Energy
A	1	−13.2	A	1	−22.0
	2	−13.2		2	−17.7
	3	−12.5		3	−14.5
B	4	**−18.0**	B	4	**−24.5**
	5	−12.9	C	5	−18.4
C	6	−12.9		6	−14.6
D	7	−12.9	D	7	−13.1

**Table 4:** Bathochromic shifts Δ*λ* in nm, molecular orbital (MO) descriptions (%) and charge transfer (CT) character of the PA1 (AH^+^)···chlorogenic acid complex (same geometry numbering as in Table 3A).

Geometry	Δ*λ*	MO	CT
1	8.03	HOMO → LUMO (0.84)	No
2	8.60	HOMO → LUMO (0.83)	Weak
3	12.03	HOMO → LUMO (0.83)	Yes
4	4.45	HOMO → LUMO (0.44)	Yes
5	0.04	HOMO → LUMO (0.63)	Yes
6	16.79	HOMO → LUMO (0.87)	No
7	7.86	HOMO → LUMO (0.85)	Yes

**Table 5:** Absorption wavelength λ in nm (oscillator strengths in brackets) of the first four excited states (S_0_ → S_i_) of the A···A PA1 dimer (same geometry numbering as in Table 2).

States	1 *	2	3	4	5
S_0_→ S_1_	451.2 (0.155)	462.1 (0.028)	452.9 (0.072)	454.1 (0.128)	468.3 (0.019)
S_0_→ S_2_	435.2 (0.093)	433.6 (0.46)9	449.1 (0.146)	450.9 (0.047)	449.3 (0.301)
S_0_→ S_3_	419.7 (0.495)	410.7 (0.019)	411.3 (0.358)	420.5 (0.649)	410.1 (0.045)
S_0_→ S_4_	405.1 (1.052)	396.3 (0.213)	399.1 (0.636)	399.3 (0.747)	393.5 (0.861)

* The spectral shifts stated in the main text are considered with respect to the absorption wavelength of A, which is 393.3 nm (oscillator strength of 0.856).

## Supplementary Materials

Supplementary materials can be found at www.mdpi.com/1422-0067/17/11/1842/s1.
