# Supplementary material for: p-Hydroxyphenyl-pyranoanthocyanins: An Experimental and Theoretical Investigation of Their Acid—Base Properties and Molecular Interactions
Source: Int J Mol Sci. 2016 Nov 5;17(11):1842. doi: 10.3390/ijms17111842 (PMC5133842; doi:10.3390/ijms17111842)
Supplement: Supplementary file 1 [file ijms-17-01842-s001.pdf]

Supplementary Materials: *p*-Hydroxyphenyl-pyranoanthocyanins: An Experimental and Theoretical Investigation of Their Acid—Base Properties and Molecular Interactions

Anna Vallverdú-Queralt, Michal Biler, Emmanuelle Meudec, Christine Le Guernevé, Aude Vernhet, Jean-Paul Mazauric, Jean-Luc Legras, Michèle Loonis, Patrick Trouillas, Véronique Cheynier and Olivier Dangles

Table S1. Chemical shifts of catechyl-pyranomalvidin-3-*O*-glucoside (PA1) (CH_3-OH-*d4*, DCl, pH1).

Position	δ ^{1}H, Multiplicity, (J_{CH} Hz)	δ ^{13}C	HMBC
C ring			
2C		161.92	H2B,H6B
3C		134.39	H9D
4C		nd	
A ring			
4aA		108.14	H6A, H8A, H9D
5A, 8aA		153.33	H6A, H8A
6A, 8A	7.13, s	101.10	
7A		167.02	H6A, H8A
B ring			
1B		120.49	H2B, H6B
2B, 6B	7.56, s	108.87	
3B, 5B		148.38	H2B, H6B, OMeB
4B		141.81	H2B, H6B
OMeB	3.92, s	57.19	
D ring			
9D	7.67, s	98.00	
10D		168.87	H9D
E ring			
1E		122.4	H5E
2E	7.53, bs	115.18	
3E		nd	
4E		nd	
5E	6.99, d, (8.6)	117.50	
6E	7.61, bd, (8.6)	123.20	
Glucoside			
1glc	4.76, d, (8.7)	104.84	
2glc	3.68, m	75.06	
3glc	3.46, t, (9.5)	77.04	
4glc	3.27, t, (9.5)	70.86	
5glc	3.17, m	77.94	
6glc	3.62, m	62.08	
6`glc	3.32, m	62.08	

s: singlet; d: doublet; t: triplet; m: multiplet; bs: broad singlet; bd: broad doublet, nd: undetermined.

Table S2. Chemical shifts of guaiacyl-pyranomalvidin-3-*O*-glucoside (PA2) (CH_3-OH-*d4*, DCl, pH1).

Position	δ ^{1}H, Multiplicity, (J_{CH} Hz)	δ ^{13}C	HMBC
C ring			
2C		162.56	H2B,H6B
3C		134.86	H9D, H1glucoside
4C		148.54	H9D, H8A
A ring			
4aA		108.74	H6A, H8A, H9D
5A		154.54	H6A
6A	7.19, d, (2.0)	101.54	
7A		168.25	H6A, H8A
8A	7.16, d, (2.0)	101.38	
8aA		153.57	H8A
B ring			
1B		120.90	H2B, H6B
2B, 6B	7.68, s	109.39	
3B, 5B		149.15	H2B, H6B, OMeB
4B		142.92	H2B, H6B
OMeB	3.95, s	57.15	
D ring			
9D	7.92, s	98.29	
10D		169.34	H9D, H2E, H6E
E ring			
1E		122.68	H9D, H2E, H5E
2E	7.65, d, (2.2)	111.48	
3E		149.86	H2E, H5E, OMeE
4E		155.07	H2E, H5E
5E	7.00, d, (8.6)	117.56	
6E	7.78, dd, (8.6, 2.2)	124.48	
OMeE	4.00, s	56.97	
Glucoside			
1glc	4.83, d, (7.7)	105.46	
2glc	3.68, dd, (9.5, 7.7)	76.01	
3glc	3.45, t, (9.5)	78.14	
4glc	3.25, t, (9.5)	71.56	
5glc	3.17, m	78.68	
6glc	3.64, dd, (11.8, 2.0)	62.57	
6`glc	3.33, dd, (11.8, 6.1)	62.57	

s: singlet; d: doublet; t: triplet; dd: doublet of doublets.

Table S3. Self-association energy (kcal·mol^{-1}) of the A···A form of PA2, according to the geometries of the different association complexes gathered in terms of structural similarities as three different groups A, B and C) as defined in text and Figure 7. The association energy of the most stable conformers is highlighted in bold.

Group	Geometry	A···A
A	1	**−32.3**
	2	−27.8
	3	−27.1
	4	−24.1
	5	−23.7
	6	−22.2
	7	−21.9
	8	−19.7
	9	−19.3
B	10	−20.0
	11	−14.1
	12	−12.2
C	13	−26.6
	14	−16.0

Table S4. Association energies (kcal·mol^{-1}) of PA2 (in both AH^{+} and A forms) with chlorogenic acid. Groups A, B, C and D are defined according to geometry similarity (see text and Figure 11). The association energies of the most stable conformers are highlighted in bold.

AH^{+}···Chlorogenic Acid			A···Chlorogenic Acid		
Group	Geometry	Binding Energy	Group	Geometry	Binding Energy
A	1	**−20.0**	A	1	**−21.3**
	2	−18.4		2	−18.3
	3	−16.8		3	−18.3
	4	−15.0		4	−16.2
B	5	−15.9		5	−16.0
C	6	−16.8	B	6	−15.8
	7	−15.1	C	7	−15.3

Table S5. Bathochromic shifts Δλ in nm, molecular orbital (MO) descriptions (%) and charge transfer (CT) character of the PA1 (A)· ·chlorogenic acid complex (same geometry numbering as in Table 3B).

Geometry	Δλ	MO	CT
1	8.90	HOMO → LUMO (0.74)	No
2	13.35	HOMO-1 → LUMO (0.83)	No
3	11.05	HOMO-2 → LUMO (0.78)	Yes
4	8.47	HOMO-1 → LUMO (0.78)	No
5	0.56	HOMO-2 → LUMO (0.51)	Yes
6	21.96	HOMO-1 → LUMO (0.76)	Weak
7	17.12	HOMO-1 → LUMO (0.75)	Yes

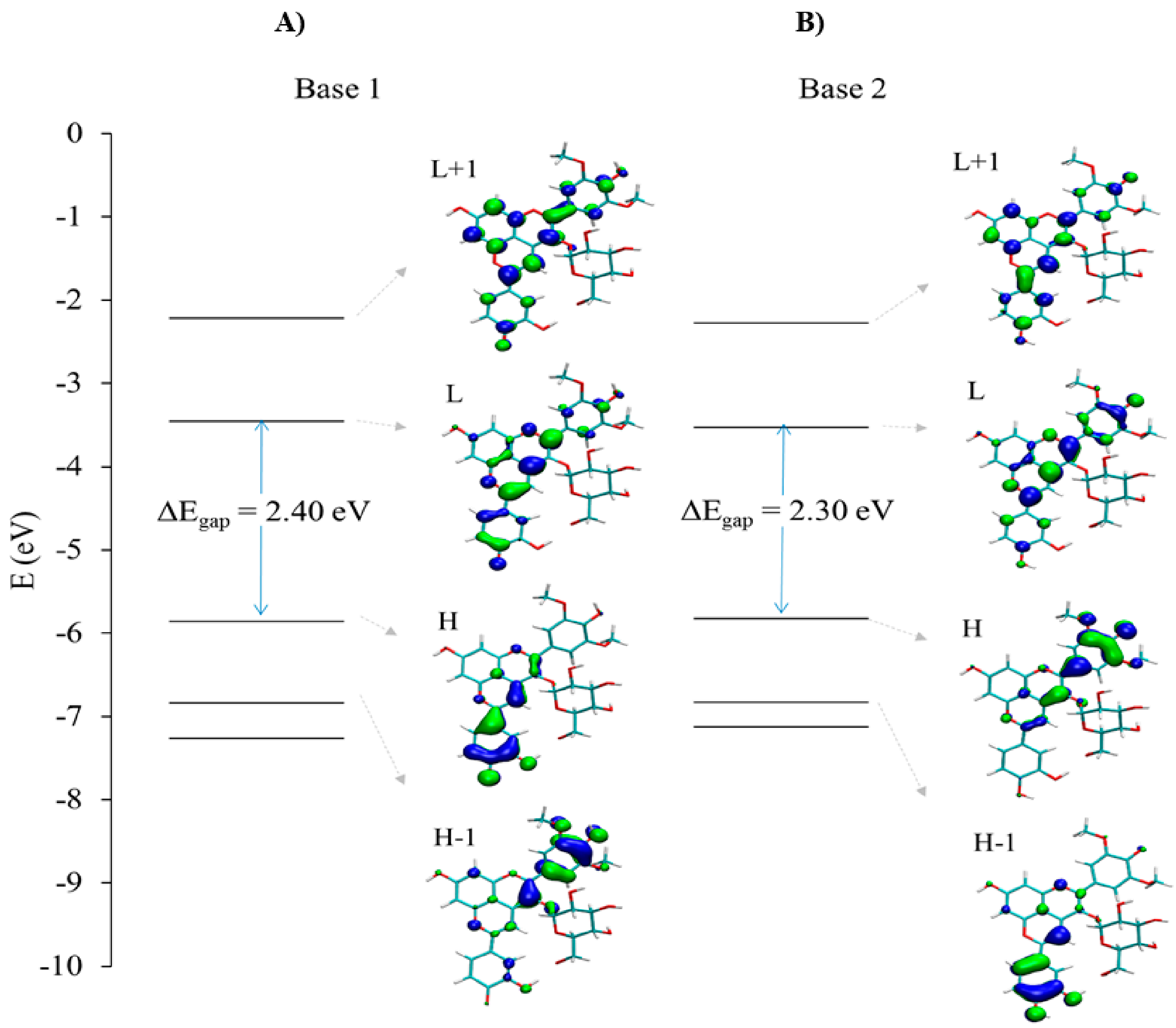

Figure S1. MO correlation diagram of PA1 for the two A forms resulting from deprotonation of (**A**) C4E-OH (base 1) and (**B**) C4B-OH (base 2).

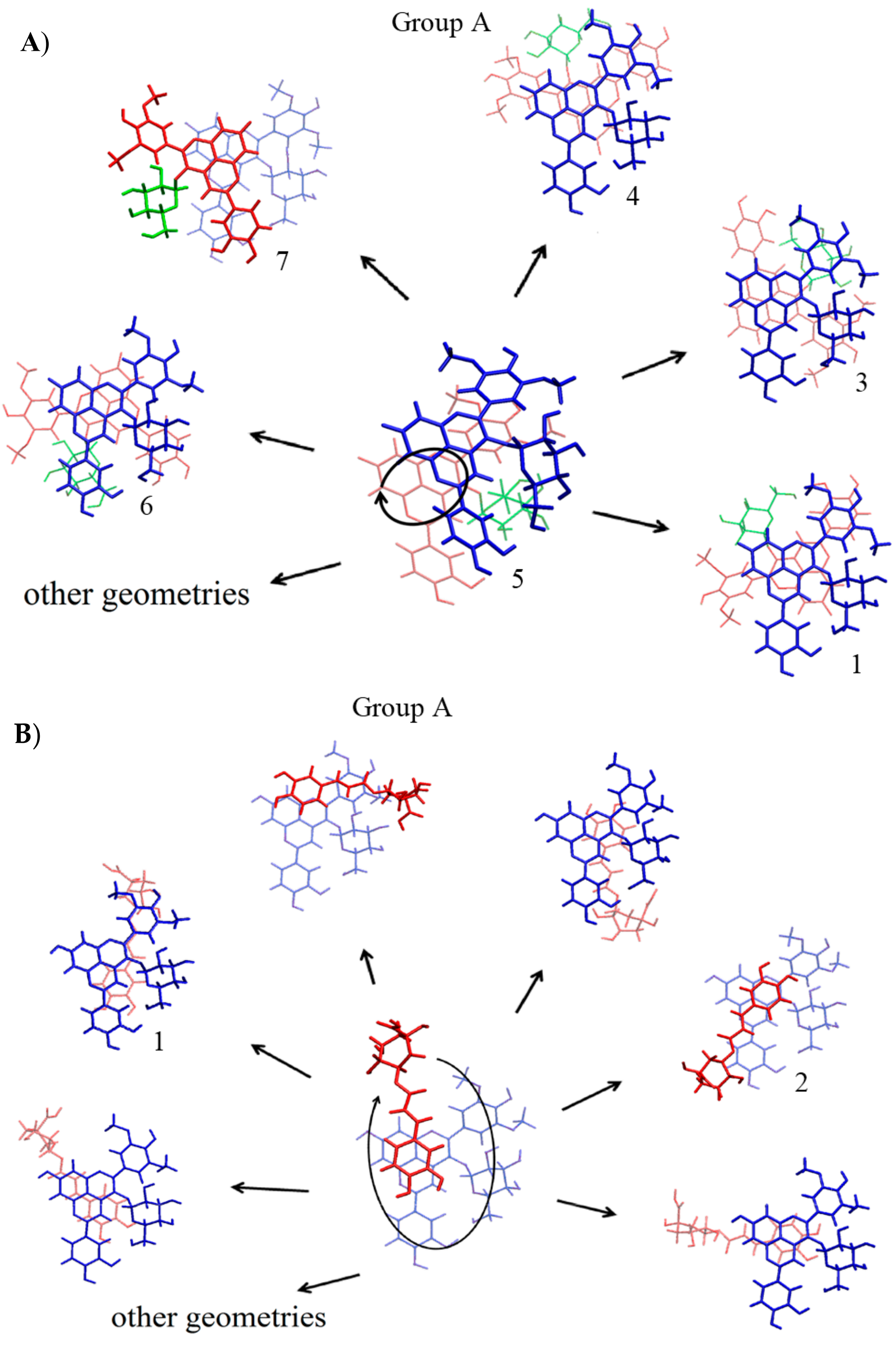

Figure S2. (**A**) Conformers of PA1 dimers with interactions between A-, C-, D- rings of both PA1 fragments. Numbers correspond to the geometry numbering in Table 2. For clarity purpose, one PA1 partner is in blue, whereas the other is both in red (pyranoanthocyanidin moiety) and in green (sugar moiety). The center structure is the structure shown in Figure 8 and it corresponds to one of the stable structures obtained for Group A; (**B**) conformers of copigmentation complexes between A-, C-, D- rings of PA1 (blue) and the catechol moiety of chlorogenic acid (red). Numbers correspond to the geometry numbering in Table 3. If there is no number, the geometry has more positive association energy than those shown in Table 3. The center structure is the structure shown in Figure 11 and it corresponds to one of the stable structures obtained for Group A. Other geometries were obtained as chlorogenic acid rotated around PA1 (shown by the circle arrow).

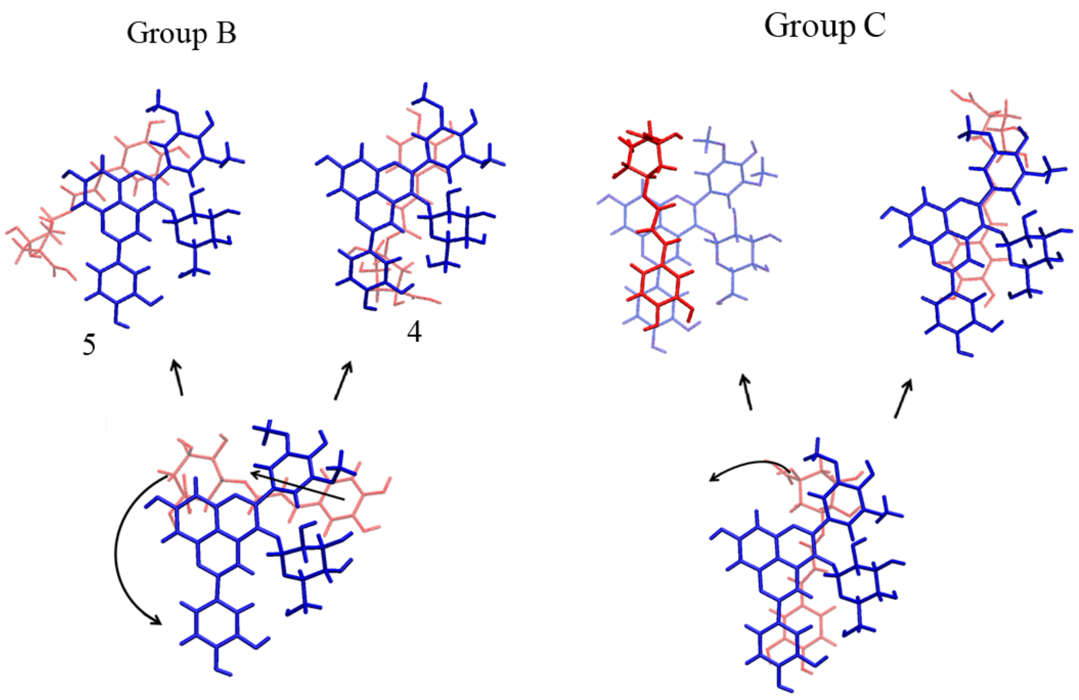

Figure S3. Conformers of copigmentation complexes between the catechol moiety of chlorogenic acid (red) and the B-ring (group B) or E-ring (group C) of PA1 (blue). Numbers correspond to the geometry numbering in Table 3. The circle arrows show the movement of chlorogenic acid along PA1.
